# Reconciling evidence-based medicine and precision medicine in the era of big data: challenges and opportunities

**DOI:** 10.1186/s13073-016-0388-7

**Published:** 2016-12-19

**Authors:** Jacques S. Beckmann, Daniel Lew

**Affiliations:** Clinical Bioinformatics, SIB Swiss Institute of Bioinformatics, CH-1015 Lausanne, Switzerland

## Abstract

This era of groundbreaking scientific developments in high-resolution, high-throughput technologies is allowing the cost-effective collection and analysis of huge, disparate datasets on individual health. Proper data mining and translation of the vast datasets into clinically actionable knowledge will require the application of clinical bioinformatics. These developments have triggered multiple national initiatives in precision medicine—a data-driven approach centering on the individual. However, clinical implementation of precision medicine poses numerous challenges. Foremost, precision medicine needs to be contrasted with the powerful and widely used practice of evidence-based medicine, which is informed by meta-analyses or group-centered studies from which mean recommendations are derived. This “one size fits all” approach can provide inadequate solutions for outliers. Such outliers, which are far from an oddity as all of us fall into this category for some traits, can be better managed using precision medicine. Here, we argue that it is necessary and possible to bridge between precision medicine and evidence-based medicine. This will require worldwide and responsible data sharing, as well as regularly updated training programs. We also discuss the challenges and opportunities for achieving clinical utility in precision medicine. We project that, through collection, analyses and sharing of standardized medically relevant data globally, evidence-based precision medicine will shift progressively from therapy to prevention, thus leading eventually to improved, clinician-to-patient communication, citizen-centered healthcare and sustained well-being.

## Background

Since the writings of the Greek physician and philosopher Galen in around 150–200 ad, healthcare has been largely influenced by organ-based anatomy. This is reflected throughout the world both in medical specialties and disease classification and in the organic structures of most hospitals [1]. The successful implementation of evidence-based medicine allowed a departure from the classic empirical practice that dominated medical history for centuries. However, it did not modify this centuries-old organ-based paradigm. Consequently, medicine deals essentially with fragmented data. Moreover, despite the broad general knowledge necessary for the practice of general practitioners and hospitalists—specialties organized around a patient and at the site of care—these experts cannot master all the required knowledge. As a result, akin to the parable of the blind men and the elephant, the fate and medical trajectory of a given patient can vary depending not only on the healthcare institution in which they are being seen, but also on which specialty portal they are first confronted with [2].

Recent technological, scientific, and social developments are likely to change this paradigm [3]. First, a flurry of revolutionary, high-resolution, high-throughput data-generating technologies keep emerging, allowing cost-effective generation of huge datasets (often referred to as “big data” [4]). Second, these developments are paralleled by continuous innovations in information sciences (such as sophisticated new algorithms and methodologies and faster miniaturized processors, sensors, and cloud computing), resulting in high-velocity and high-capacity computation facilities. The third major factor is embodied by the patients or citizens themselves. Individuals, empowered by the proliferation of social media (see, for instance, [5, 6]) and connected technologies and electronic devices [7, 8], manifest a growing will to participate in managing their own health and to also interact with others afflicted with similar diseases. While each of these developments is disruptive on its own, together they ensure that we are living in perhaps one of the most profound periods of advancement in biology and medicine, leading to a medical revolution that will contribute to precision medicine [3] and transform health and medicine.

The long-term goals of precision medicine are numerous. They include better disease delineation and stratification, detection and monitoring of disease symptoms as early as possible, identification of presymptomatic individuals (that is, (long) before the disease is clearly manifest), monitoring and modeling the dynamics of disease evolution, and improved surveillance and management of disease. Prominent among these goals are to provide better-adapted, personalized surveillance measures and therapies and to significantly delay disease onset and, whenever possible, to prevent it. Consequently, it can be envisaged that the main focus in healthcare will progressively shift—in a safe, efficient and cost-effective manner—from treating disease to managing health. Realization of these ambitious objectives will result in significantly improved health outcomes and patient satisfaction overall.

To achieve these overarching goals, it will be necessary to bridge current evidence-based medical practice with precision medicine (Box 1) and share, in a standardized format and fashion, data across centers and countries. Here, we outline our view of the challenges that need to be surmounted to transform these opportunities into real clinical benefits allowing the practice of evidence-based precision medicine.

**Table 1:** Examples of challenges and opportunities of evidence-based precision medicine

Challenges	Opportunities
•Multiplicity of stakeholders and disciplines •Analyses of big data •Heterogeneity of complex, multilayered data types, and formats •Harmonization of data semantics (clinical, laboratory, and others): vocabularies, terminologies, classification and coding systems, ontologies •Standardization of data entry and storage •Integration of multiple data types (such as laboratory, clinical, behavioral, lifestyle, environmental) •Secure, sustainable, and effective data storage and sharing •Necessity for new analytic tools and algorithms •Multiplicity and lack of semantic and technical interoperability of electronic health record systems •Extremely dynamic and fast-changing field, with new tools constantly emerging •Training and education of the different stakeholders (medical staff, patients, and decision-makers) •Ethical, legal, social, and consent issues •Uberization of medicine	•Improved disease delineation, classification, and stratification •Detection and monitoring of disease symptoms as early as possible •Non-invasive prenatal or cancer testing •Identification of pre- or asymptomatic individuals •Identification of new disease mechanisms and treatment modalities •Monitoring and modeling the dynamics of disease evolution •Improved, personalized surveillance and management of disease and therapies •Significant delay of disease onset and, whenever possible, prevention •Development of evidence-based precision medicine •Shifting emphasis of medicine more from therapy to prevention, and from disease to wellness •Systemic view of medicine •Patient participation •Patient-centered medicine

## The role of clinical bioinformatics in precision medicine

The capacity to produce and interpret the wealth of data produced by these technological and scientific innovations has already profoundly modified the scientific landscape, including our understanding of biology, and will continue to do so [9]. This has allowed us, for instance, to sequence the genomes of hundreds of thousands of individuals, to determine the allele-specific compositions and relative abundances of RNA transcripts in numerous cell types and conditions, to explore protein [10] or metabolite [11] profiles, to reconstitute molecular, cellular or physiological mechanisms, pathways and networks, and thus to generate new testable scientific hypotheses.

These developments are also likely to impact medical practice. The wealth of data (both multi-scale and multi-level), which is theoretically already available for any given individual, requires increasingly complex, sophisticated, multidimensional analyses in order to convert these heterogeneous, large-scale datasets into clinically useful information. This is where clinical bioinformatics (an essentially multidisciplinary approach whose key functions include the utilization and integration of laboratory and clinical data and the use of databases, computational methods, algorithms, and other resources and methods) enters the arena as an essential element of data-driven precision medicine.

There are numerous definitions of clinical bioinformatics or related terms (for example, see [12]). Our perception of what this field entails is that by enabling the bioinformatics mining and use of “omics” and other high-throughput data in a clinical setting, by integrating various standardized and interoperable datasets, by extracting valuable clinically useful medical knowledge from these data resources, and by providing clinical-grade analyses or decision-support tools, clinical bioinformatics bridges the gap between medical practitioners and the fruits of biological research. Hence, as medicine tends progressively to be more and more intensively data driven, clinical bioinformatics aims to support diagnosis as well as tailored preventive and therapeutic approaches in order to facilitate a personalized approach to health.

## Big data and clinically useful knowledge

It is not our purpose here to review these high-throughput data-generating technologies, their limits, nor all the potential they offer. It suffices to know that they allow the generation of complex, heterogeneous big data (such as high-resolution molecular omics, imaging, clinical, and other, emerging data types), also referred to as a “digital phenotype” [13]. Integration and analyses of these multiple types of data sets, which include data ranging from single cells [14, 15]—or parts thereof—to organs, entire organisms, or populations, as well as including data on individual lifestyles (for example, [16]), environments, or social media (for example, [6, 13, 17, 18]), will provide high-level views of exquisite granularity. It is logical to expect that this ever-growing amount of high-resolution data generated by these transformative tools can and will lead to a profound shift in healthcare. This will be contingent upon the data being eventually translated into clinical benefits for patients as well as for individuals at large—that is, be useful at the point of care as well as at the population level [19].

This vision has led to a refinement of the “classic” concept of precision medicine, also referred to as personalized medicine [20]—in our opinion, the terms “precision”, “personalized”, and “stratified” medicine are essentially equivalent and interchangeable, with preference for one or the other reflecting more a matter of fashion or national preference. Analyses, interpretation, and exploitation of this wealth of (longitudinal) data (across cell types, organs, tissues, individuals, cohorts, lifestyles, or environments), embodied in activities regrouped under the term “clinical bioinformatics”, are likely to provide unprecedented opportunities for integrative approaches, allowing a shift from the traditional organ-based paradigm to a more all-inclusive and systemic assessment of health and disease, and the practice of systems medicine [21–23].

It is important to stress that improved understanding and management of health-related issues, or any complex biological situation, is best served by systemic explorations. By “complex”, we refer to situations where the whole is more than the sum of its constituent parts. The latter can be intertwined and interrelated, forming complex networks, and the resulting intricacies are often not visible when these components are investigated individually—that is, out of context. However, the utility of reductionist approaches should not be downplayed. Quite the opposite. Reductionist approaches, by providing the elements required for the understanding of the components of the larger system, can be, and have been, incredibly successful, having paved the way to the present scientific and medical knowledge. Yet, deconvoluting this complexity to consider only its separable components will often fail to capture the interactions between all components (the “interactome”) and thus the complexity of the entire unit, and therefore seriously limit our capacity to understand, treat, or prevent diseases. Therefore, both reductionist and integrative approaches are necessary as they complement each other. In medicine, this complexity pertains not only to the individual as a whole, but should also include his/her environment (or “exposome”) and behavior. We offer below a selected set of examples of achievements made thus far.

### Genomic sequencing

With the continuously decreasing costs of next-generation sequencing (NGS), much attention has been directed towards clinical implementation of NGS of whole genomes or fractions thereof. As a result, a number of low-hanging fruits of NGS are already regularly collected and used [24], resulting increasingly in actionable therapeutic insights. After the widespread implementation of genetic diagnoses for Mendelian diseases, NGS of circulating fetal- or tumor-derived cell-free plasma DNA are prominent and successful examples of non-invasive diagnostic applications that are being used, respectively, for prenatal diagnosis of fetal aneuploidies (for example, [25, 26]) or cancer diagnosis (for example, [27–29]). Similarly, NGS of cancer cells or tumors can suggest potential therapeutic leads (for example, [30–35]). Another telling clinical sequencing application is in the area of microbial pathogen identification or for the epidemiologic analysis of pathogen spread and evolution, thereby opening new avenues for timely therapies, infection control, and public-health responses (for example, [36, 37]).

As cost-effectiveness increases, NGS is fast moving from being essentially a research tool to being adopted into clinical practice. These examples illustrate the power of NGS-based analyses, which are likely to become increasingly part of routine screening in this fast-evolving landscape. Some advocate that, in the foreseeable future, every individual could have their genome sequenced (see, for instance, [38]), at least once during their lifetime. This will, however, require not only further substantial decreases in sequencing costs, appropriate management of potential iatrogenic harm, such as caused by the “incidentalome” (Box 1) [39, 40], but also new methods to convey the information back to the tested individuals with effective counseling and coaching strategies [41].

While most attention is currently focused on DNA sequencing [42], it should be emphasized that, however valuable and informative our genomic DNA sequences can be, our genomes do not encapsulate all the needed information that determines our health status as not everything is written in our genomes. While the genotype-first approach [43] is attractive, the road from genotype to phenotype is loaded with caveats and uncertainties [44]—consider, for example, factors such as incomplete penetrance, the description of clinically discordant monozygotic twins (as reported, for instance, among carriers of the ∂508 mutation in cystic fibrosis [45]), the fact that a given mutation can result in clinically distinct disease entities (see, for instance, [46–48]) or can lead to variable pleiotropic effects [49], the description of resilient “superheroes” [50, 51], the difficulties of interpreting variants of unknown significance [52] or even of estimating the relative risks of known pathogenic mutations [53], and the missing heritability [54]. Furthermore, besides germ-line variants, somatic mutation events may also need to be considered, even beyond cancer, given the finding of somatic mosaicism [55] and Venter and colleagues’ assertion of the “dynamically changing nature of our genomes throughout life” [56]. The detection and monitoring of such dynamic changes will require more than one sequencing test. These are but a series of examples demonstrating the current limitations in the proper interpretation of known genomic sequence variations, both for monogenic entities and even more so for complex diseases [19, 44].

### Inherited and acquired elements

Predictive power will certainly improve with increased integration of ancillary genome-derived information, such as epigenetics and expression data (which we refer to below as “meta-omics”). However, not every aspect of health is determined, directly or indirectly, by the genome inherited from our parents. The germline genome is an essential component, but is only one of several layers of information.

This is best illustrated with information that so far has only just begun to enter the medical arena. In the past decade, there has been an increasing awareness that, besides our own inherited genome, each individual hosts “alien” genes (our microbiome or metagenome), with perhaps up to tenfold more microbial cells than human cells, and the collective number of microbial protein-coding genes (and thus gene complexity) is orders of magnitude larger (close to 500 times) than that encoded by our nuclear genome [57, 58]. Our microbiome, which also contributes to a significant fraction of our metabolome [59], assumes essential functions in regulating growth and homeostasis. Its composition is influenced by our own Mendelian genome [60], and yet no two individuals are alike—that is, even monozygotic twins might not share the exact same microbiome [60–63]. In addition, while our inherited genome is, but for a few exceptions, relatively static throughout life, our microbiome is considerably more dynamic (for example, [64–66])—its landscape changes and fluctuates in response to a number of intrinsic and external factors (such as disease, antibiotic therapy or other medications, age, diet, lifestyle, socioeconomic status, and geography). This plasticity endows the host with the capacity to rapidly adapt and adjust to changing environments [67] and to face adverse or stressful conditions (for example, [65, 66]). In other words, the microbiome is a sensor for the extrinsic and intrinsic ecosystem in which we dwell and evolve; it can thus be seen as an essential element, among other factors, ensuring the robustness needed for survival in rapidly changing, and potentially adverse, environments. The microbiome has also been causally implicated in numerous diseases [65, 68], including brain diseases (for example, [69–71]), as well as in response to therapies [72, 73]. Hence, besides our inherited genome, the microbiome could also be a major player in disease and well-being, and this might justify that its composition should become, in the foreseeable future, part of routine clinical screening programs, allowing for a more comprehensive understanding and management of individual health.

Proper consideration of this complex multicomponent partner—an integral element of our hologenome [67, 74], which, in addition to our own genome, includes our microbial flora as well as a largely unexplored viral component [75]—will enable a more global approach to human health. However, given the dynamic plasticity and complexity of the microbiome, the data needed to capture this information throughout an individual’s lifetime might be orders of magnitude larger than those required for our nuclear genome. This contrast is further exacerbated if all associated meta-omics data are included.

While interest in the impact of the microbiome on health has grown tremendously over the past few years, most of this work still resides in the realm of basic science. Potential clinical implementations are often emphasized, but their realization requires further consolidation and validation. Recent data suggest that anticancer therapies might need to consider each patient’s microbiome as the latter might shape immune-surveillance and autoimmunity and might significantly affect the metabolism of anti-cancer agents [76, 77]. If further substantiated and characterized, this area could have a significant impact both on prophylaxis as well as on treatment of cancer. It is also tempting to speculate that oral decoctions in traditional Chinese medicine specifically target the hologenome [78–80], but further studies are necessary to clarify this point.

### Integrating other data types

In addition to the inherited and acquired factors contributing to the health of an individual, consideration of a given cell type, tissue, or organ can now be envisaged longitudinally, integrating the available information over time and space with that of other body cells, tissues, or fluids. Furthermore, the same tools provide the opportunity to also take into account data on the environment, as well as on individual activities, behavior, or social networks. This will allow us to progressively depart from anatomy-based medicine to eventually enable a more all-inclusive, systemic assessment of a given individual’s health, considering the whole rather than its constitutive parts and leading to the development of what others have called “P4” (predictive, preventive, personalized, and participatory) medicine [81] targeting the optimization and prolongation of wellness [82].

Indeed, being able to explore detailed datasets, including those contributed by individuals themselves, should enable more comprehensive, global (systemic) views of their health status and thus enable both earlier and more accurate diagnoses, as well as more appropriate, customized therapeutic interventions, and thus contribute to the development of P4 medicine.

## Aggregation of heterogeneous data sets into electronic health records

To achieve these objectives, these data, which are of multiple heterogeneous types, formats, and sources, must be integrated among themselves and with each individual’s phenotypic and clinical records. The latter are currently stored in electronic health records (EHRs). Yet, with over 700 distinct EHR vendors as well as numerous in-house developments, medical data entry, database management, and other processes can vary both from vendor to vendor and from hospital to hospital. Hence, the market is alas fragmented, often creating what amounts to “EHR silos” [83]. Furthermore, storage and analysis of large data sets are not easily achieved using current EHRs, resulting in a growing trend to use ancillary data stores, such as picture archiving computer systems (PACS) for imaging and emerging warehouses for genomic data. Medical records remain extremely heterogeneous, and integration of data across EHRs can be daunting as current EHRs are neither always interconnected nor mutually compatible or interoperable.

This point is well summarized by the Community Research and Development Information Service (CORDIS) who stated that: “The clinical domain is probably among the most complex from a semantic point of view. Vocabularies, terminologies, classification and coding systems, and ontologies have been developed by different stakeholders to address different needs in different subdomains” [84].

There is a clear necessity for robust standardized procedures, formats, structured data, and nomenclature ontologies to ensure reliability and efficiency [85]. However, EHRs are not only heterogeneous but they can have incomplete, inconsistent, or inaccurate data, as well as additional limitations (reviewed elsewhere [86–88]) that further hinder their applicability, scalability, and semantic and technical interoperability. These issues can represent major obstacles towards the efficient implementation of precision medicine as the extraction of robust medical information can often require cross-EHR meta-analyses, justifying the call for common standards [89], as emphasized below.

## Reconciling evidence-based medicine and precision medicine

The paradigms of evidence-based medicine and precision medicine both have strengths and weaknesses. In evidence-based medicine (Box 1), data are collected from populations or large cohorts, from which mean values or figures are derived to infer recommendations [20], which will be applied to all, approximating the “one size fits all” scenario. In general, outliers are essentially ignored. Although, for most traits, each person might fall within the mean estimates of evidence-based medicine, any individual is nevertheless likely to be an outlier for one or several conditions for which they might be a poor responder to the recommended evidence-based medical practices. Under these circumstances, evidence-based medicine might fail to provide an adequate response.

This stands at odds with precision medicine, which focuses on the individual, for which massive data-points have been collected. However, precision medicine also has its limitations. With millions of data-points per individual, each case might be so particular or unique that one might increasingly face “N-of-one” situations. In view of the data scale and high level of complexity, this can considerably limit the statistical power required to set up and define appropriate evidence-based guidelines, even if, for some patients, detailed functional investigation might resolve this problem. Thus, the difficulties in discriminating between significant and anecdotal inferences based on “N-of-one” situations also creates a medical problem. At best, the individual might occasionally serve as their own proper control [90].

For medical practice to increasingly shift from generic (“one size fits all”) to precision and personalized treatment, there will be a need to reconcile evidence-based medicine and precision medicine as, despite their respective limitations, they can and should be viewed as mutually complementary. The added value gained by merging the strengths of both approaches relies on our capacity to perform deep investigations of large cohorts of patients. Indeed, the conversion from single cases to an evidence-based approach will imply collation and meta-analyses of big data from cross-institutional and transnational large-scale registers and cohorts [91]. This will allow detailed analyses of these very large population samples, allowing, whenever possible, aggregation of data from similar “N-of-one” cases, resulting in “N-of many” paradigms, so that robust, reliable inferences can be drawn for these stratified subgroups [90]. This transition to an evidence-based precision medicine will, however, necessitate standardization as well as responsible sharing and mutualizing across numerous interoperable data warehouses. Following the same rationale, the numerous registries and large biobanks that are assembled all over the world should also be constructed in such a way as to warrant this need for inter-operability.

Thus, to safeguard optimal connectivity and utilization of the vast amounts of data that can be collected in each of these invaluable biobanks or clinical resources, it is essential to ensure their accountability, transparency, compatibility, and harmonized interoperability. To enable access to this massive data set, it will be necessary to create sustainable federated, safe data commons or warehouses that will, under the appropriate conditions, be open and available to all, thus facilitating responsible data curation and sharing while duly respecting all ethical and legal concerns and fully protecting the privacy of the contributors [92]. Adequate solutions need to be developed to meet these ambitious goals. Hopefully, through federated efforts such as the Global Alliance for Genomics and Health [93], these issues will be resolved, allowing optimal use of these data for research (for example, in disease modeling) as well as in clinical care.

## Citizen-centered medicine

The patients or citizens embody an additional central element of these quantitative as well as qualitative transformations of healthcare. Active patient involvement will not occur overnight. It might require adequate sensitization and education, but will hopefully progressively settle in as patients gradually and presumably irreversibly shift from their ancient position of passive subjects to being active as well as proactive participants and managers of their own healthcare. This transition is enabled by the distribution and diffusion of, and widespread access to, comfortable, inconspicuous, user-friendly miniaturized connected mobile devices or other wearable or implantable sensors (among other technologies) that capture (often cheaply, automatically, effortlessly, and continuously) geolocation, pollution, environmental, behavioral, lifestyle, physiological, or other clinically relevant data. This evolution has already prompted the burgeoning fields of mobile health (“mHealth”) and quantified self, opening the way to mobile-device-based non-invasive (self-) diagnosis and real-time or remote monitoring [7, 8]. This might also contribute to a transition from a classically paternalistic approach in medicine to patient- or consumer-driven healthcare.

These devices offer new and exponentially expanding opportunities and challenges in numerous areas, including the health ecosystem. New mobile health applications are constantly appearing (over 100,000 were available by the end of 2014 [94, 95]), including applications for routine laboratory tests, such as for blood chemistry. For these quantitative and qualitative data to be of clinical utility, it will be necessary that mobile health applications and devices be properly evaluated and approved for the utility, safety, quality, accuracy, and reliability of the collected information [96]. With increased connectivity, use of certified connected wearables and smart implants could allow cheap, continuous, longitudinal harnessing of vast amounts of high-quality clinically useful data, allowing the collection of large-scale biologic, personal, environmental, and social information. In the not-too-distant future, these processes are likely to result in a “medico-sphere” in which patients become big data producers so that more medical data will be present in individuals’ smartphones (or other devices) than in their EHRs. This could lead to a major medical as well as social shift in healthcare, as people take an increasing role in their own health management.

While these applications might prove extremely useful in several fields, such as in redesigning and evaluating clinical trials or in critical care medicine [97], medical knowledge might no longer be the prerogative of the healthcare profession. Optimal use of this information will still rest on the competences and skills of the medical profession. However, with medical care progressively becoming individual-driven, patients could increasingly assume responsibilities for their own health, resulting in a democratization of healthcare. The citizens could own and control most of their personal medical data, which could essentially no longer belong to the hospitals. This could also impact the relationships between individuals and their physicians, as the latter might turn increasingly into a commodity or service, dissociated from hospitals, with individuals rating, and selecting, their physicians. This “uberization” of medicine [98] is thus likely to impact current models and standards of healthcare practice.

Furthermore, access to relevant, specialized medical information will be easily enabled via the internet, which will be further amplified via social media, including disease-centered patient groups [6]. The capture of personalized health-related records might incentivize individuals to stay healthy. They might be able to monitor vital parameters and, by proper lifestyle adjustments, to reinforce positive behavior and to improve medical compliance and thus maintain or ameliorate their general health status. This might result in increased quality-of-life and life expectancy. This could also facilitate “health coaches” to intervene with adequate personalized advice. Together, this might result progressively in increased citizen empowerment and self-management.

Moreover, citizens might not only be actively engaged in their own healthcare, they might also have the opportunity to become research partners. With appropriate regulation and consent, they might (and hopefully will) opt to contribute their self-collected health-related data to biomedical research (for example, [99, 100]), thereby supporting citizen science. This would, once again, necessitate (i) careful design of public data collection, curation, storage, sharing, in due respect of the participants’ cultural sensitivities, privacy and consent, and possibly control, and (ii) that the healthcare system be able to integrate and use these data. Patient-centered empowerment is thus likely to affect healthcare dramatically.

## The economics of precision medicine

Another essential driving force in precision medicine is health economics. While combining evidence-based medicine and precision medicine approaches will optimize medical practice, there is a danger that this might also signify the end of the era of the development of blockbuster drugs, inasmuch as precision medicine focuses on small, stratified sub-populations. In other words, precision medicine pertains essentially to small niches. Considering that there is already the problem of the high cost of new medications, the ensuing reduction of marketing niches could lead to serious economic and social problems, as costs required for the development and validation of new drugs, and thus their selling value, might become prohibitively expensive. We surmise that, despite this risk, the overall returns will shift the balance towards important savings for healthcare. This rests on the assumption that, although new niche medications will continue to appear, the major benefits will not be in the area of treatment but, as stated earlier and elsewhere [82], in disease prevention or retardation.

In our opinion, the major goal of precision medicine is not to extend life expectancy (although this is a likely byproduct), but to improve long-term wellness. Diminishing disease severity considerably or, even better, delaying disease onset by one or several years (or almost totally) are the major benefits to be gained from this precision medicine paradigm. Moreover, this gain should be readily implementable in all parts of the world, thereby contributing to a global democratization of healthcare. According to this scenario, medicine will continue to rely on adjusted and personalized treatment, but the major goal of precision medicine will focus on increased well-being.

## Challenges of evidence-based precision medicine

The challenge of understanding the underpinnings of our health in increasing detail can be overwhelming, and there could be significant potential pitfalls. The field of precision medicine should advance with caution, avoiding overselling and ensuring that any claim made rests on solid grounds. The challenges ahead are numerous, but so are the rewards—namely that each and every individual should eventually be able to benefit from precision medicine (Table 1). Probably no other medical field has ever progressed so rapidly. Consequently, this has ramifications that are likely to impact many aspects of the entire medical domain.

To begin with, it will call for adequate educational and training efforts and might necessitate frequent revisiting and adjusting of educational programs to adequately reflect the existing but constantly evolving state-of-the-art of precision medicine. As the European Science Foundation asserted: the “healthcare profession may, as a result, need to undergo a radical overhaul” [101]. It is not unlikely that, in a couple of years, we will see, akin to what happened in the fields of radiology and imaging, the increasing importance of the medical specialty (and possibly sub-specialties) of clinical bioinformatics. This evolution might require a profound reshaping of medical curricula to train highly specialized experts, responsible for the interpretation of the ever-increasing amount and complexity of data, for modeling disease (onset, progression and treatment) and for issuing reports that will serve as decision support for clinical staff. However, this educational issue concerns all other healthcare stakeholders as we will need to ensure, on the one hand, the proper education and training of experts in clinical bioinformatics and, on the other, the familiarization of all concerned medical stakeholders (MDs, nurses, technologists, lab technicians, pharmacists, and other medical or clinical staff) with this specialty. Or, as stated elsewhere, “future education and training must reflect the changing body of knowledge and must be guided by changing day-to-day informatics challenges” [102]. The days when a single physician (or even hospitalist) could cover the entire gamut of medicine are gone, as the practice of medicine becomes increasingly multidisciplinary. The field of clinical bioinformatics is likely to evolve rapidly and continuously, and will do so in an unprecedented manner. To capitalize best on these rapid advances, the corresponding future training curricula will need to be reassessed and readjusted periodically as well as adapted regularly for the continuous training of practicing physicians and other healthcare professionals.

Given the potential future role of citizens in self-management of health as part of precision medicine, it is also important that adequate attention and resources are allocated to promote awareness of personal health and to keep the public adequately informed of the status of the field and how best to benefit from it. Keeping the public sector abreast of the field is a huge but necessary endeavor. It is essential that the public is involved in this transformative process as engaged partners rather than as bystanders. Doing so will further incentivize them to participate in this medical revolution. Only then will they be able take full benefit of all the opportunities afforded by this democratization of healthcare.

## Conclusions

We consider that evidence-based precision medicine rests on three pillars: (i) responsible inter-institutional sharing of large clinical and laboratory interoperable, harmonized data sets; (ii) data on vital signs and behavior collected by empowered citizens; and (iii) clinical bioinformatics required to convert this complex information into clinically useful knowledge, which will be returned by the medical practitioners to the individuals concerned. As it becomes progressively easier to collect huge amounts of disparate personal health- and population-related information on a global scale, the real challenge in clinical bioinformatics will be to curate, store, federate, integrate, share, mine, interpret, and transform these extensive heterogeneous data into scalable, medically actionable resources, while paying due respect to all legal, ethical, and privacy values [103, 104]. This could allow the transition from reactive to proactive medicine. In other words, this knowledge could lead us to revisit disease etiologies, to refine, stratify, or reclassify diseases, and to identify new disease mechanisms and treatment modalities. The net outcome could be better clinical diagnosis or prognostication; this could facilitate clinical decision-making, improve medical care or treatment, and most importantly, could contribute to disease delay or even prevention. Clinical bioinformatics has a central role to play in this revolutionary person-centric effort by contributing to care delivery innovations and improved health preservation, and shifting the emphasis more and more from therapy to prevention, and from disease to wellness.

## Box 1 Glossary

**Table:** 

Clinical bioinformatics	Bioinformatics mining and use of omics and other high-throughput data in a clinical setting, integrating various standardized and interoperable datasets, extracting valuable clinically useful medical knowledge from these data resources, and providing clinical-grade analyses or decision-support tools.
Clinical utility	The relevance and utility of an intervention in patient care; the likelihood that an intervention will improve patient outcomes [105].
Evidence-based medicine	The use of evidence from well-designed and well-conducted research (such as from meta-analyses, systematic reviews, and randomized controlled trials) to optimize decision-making in medicine [106].
Electronic health record (EHR)	Digital version of data pertaining to the health status of patients (such as medical and treatment histories), and allowing easy and secure information retrieval.
Incidentalome	Ensemble of abnormal secondary incidental findings.
Interoperability	Ability to exchange electronic information, based on implementation of standards, without special effort on the part of the user.
Metabolomics	The high-throughput identification and quantification of small-molecule metabolites or exogenous substances present in cells, tissues, biofluids, and organisms.
Microbiome	The collective genome of our indigenous microbes present in a biological specimen or organism.
P4 medicine	Acronym referring to predictive, preventive, personalized, and participatory (P4) medicine, a systems approach that is proactive and individualized, with an emphasis not only on disease, but also on wellness [23].
Personalized medicine	Medical interventions tailored to a specific patient based on the individual characteristics of this patient and their inferred response or risk of disease.
Precision medicine	Precision medicine seeks to move away from symptom-based taxonomies towards the development of individualized care, to be achieved through the molecular characterization of individuals in a multi-layered patient-centered system, with customized medical interventions, taking into account a myriad of factors (such as the patient’s genome, environment, and lifestyle) that can influence development of disease or treatment response and thereby improve health (modified from [101]).
Quantified self	Self-monitoring and data acquisition on, among others, vital signs, behavior, and lifestyle, as a means to improve health and fitness.
Stratified medicine	While there may be subtle differences in the literal meanings of the terms “personalized medicine”, “precision medicine”, and “stratified medicine”, they usually refer to the same concept when applied in practice. Stratified medicine (mainly used in the UK) is more treatment-dependent, targeting it according to relevant (biological, clinical, and other) characteristics of subgroups of patients [107].
Systems medicine	Interdisciplinary study of the systems of the human body as part of an integrated whole, incorporating biochemical, physiological, and environment interactions [108].

## Abbreviations

CB: Clinical bioinformatics
CORDIS: Community Research and Development Information Service
EHR: Electronic health record
MD: Medical doctor
mHealth: Mobile health
NGS: Next-generation sequencing
P4: Predictive, preventive, personalized, and participatory
PACS: Picture archiving computer systems
